# Traumatic Experience and Somatoform Dissociation Among Spirit Possession Practitioners in the Dominican Republic

**DOI:** 10.1007/s11013-015-9472-5

**Published:** 2015-10-01

**Authors:** Yvonne Schaffler, Etzel Cardeña, Sophie Reijman, Daniela Haluza

**Affiliations:** Department of General Practice, Center for Public Health, Medical University of Vienna, Kinderspitalgasse 15, 1090 Vienna, Austria; CERCAP, Department of Psychology, Lund University, Lund, Sweden; Institute of Environmental Health, Center for Public Health, Medical University of Vienna, Vienna, Austria

**Keywords:** Spirit possession, Somatoform dissociation, Traumatic experience, Dominican Republic

## Abstract

Recent studies in African contexts have revealed a strong association between spirit possession and severe trauma, with inclusion into a possession cult serving at times a therapeutic function. Research on spirit possession in the Dominican Republic has so far not included quantitative studies of trauma and dissociation. This study evaluated demographic variables, somatoform dissociative symptoms, and potentially traumatizing events in the Dominican Republic with a group of *Vodou* practitioners that either do or do not experience spirit possession. Inter-group comparisons revealed that in contrast to non-possessed participants (*n* = 38), those experiencing spirit possession (*n* = 47) reported greater somatoform dissociation, more problems with sleep, and previous exposure to mortal danger such as assaults, accidents, or diseases. The two groups did not differ significantly in other types of trauma. The best predictor variable for group classification was somatoform dissociation, although those items could also reflect the experience of followers during a possession episode. A factor analysis across variables resulted in three factors: having to take responsibility early on in life and taking on a professional spiritual role; traumatic events and pain; and distress/dissociation. In comparison with the non-possessed individuals, the possessed ones did not seem to overall have a remarkably more severe story of trauma and seemed to derive economic gains from possession practice.

## Introduction

An important question in the research of spirit possession is why certain individuals experience possession and others do not. Exposure to traumatic events and stressful events, dissociative tendencies, and somatization have been discussed as predisposing factors (Igreja et al. 2010; Neuner et al. 2012; Sapkota et al. 2014; Seligman 2005a; Van Duijl et al. 2010). Whether spirit possession necessarily involves some kind of dysfunction or psychopathology has been the theme of a long-lasting scientific debate (Boddy 1994; Owen 1990). In this discussion, anthropologists have tended to argue that spirit possession is a culturally sanctioned and rewarded phenomenon that fulfils a social function and needs to be interpreted within its cultural context (Boddy 1988; Crapanzano 1977; Lambek 1989). Other authors, mostly from psychological and medical disciplines, have described spirit possession cases associated with chronic affliction and dysfunction, sometimes as a cardinal, sometimes as a secondary symptom of a pathological syndrome such as psychosis, somatization, or a dissociative disorder (e.g., Gaw et al. 1998; Goff et al. 1991; Ng 2000; Somasundaram et al. 2008; Ward and Beaubrun 1981; for a review see Cardeña et al. 2009). In either case, it may be too simplistic to assume that there is only one path towards the development of dissociative tendencies. For instance, high hypnotisability is differentially related to high punishment/trauma on the one hand, and the development of a rich fantasy life on the other (Hilgard 1970). It is also simplistic to assume that possession is exclusively either functional or dysfunctional. In this vein, Somasundaram, Thivakaran, and Bugra (2008:249) found that possessions had been of financial benefit to 43 % of experients within recognized community groups, while about 47 % of psychiatric patients who experienced prolonged and uncontrollable possession reported having suffered financially.

Based on a questionnaire- and interview-based survey, our study evaluated factors related to *Vodou* possession in the Dominican Republic, comparing possessed individuals to a group matched in terms of socioeconomic status (SES) and religious belief. Possible predictors of possession included traumatic experiences, somatoform dissociation, environmental and familial influences, and some aspects of inner experience. Moreover, we explored whether or not possession was associated with chronic affliction or, contrariwise, with personal or social benefits.

### Definition

The notion that a person’s ordinary identity may be replaced by a different one, usually that of a spiritual entity, is commonly referred to as spirit possession. Spirit possession typically results in noticeable changes in consciousness and behaviour followed by reported amnesia for the event. Variants of it are found throughout history and the globe (Boddy 1994; Bourguignon 1973; Oesterreich 1921/1974). Spirit possession is one of a number of alterations in the sense of self and identity (Cardeña and Alvarado 2011), sometimes discussed under the term of ecstatic religions and referred to as *central possession* when the alterations are part of a culturally sanctioned set of beliefs and practices, or *peripheral possession* when they occur independently or at the margins of cultural or religious practices (Lewis 1971/1989). In this paper we refer to spirit possession as the phenomenon, as evidenced in behaviour and experience, of one identity being replaced by another, and not to the explanation of diverse phenomena such as illness being caused by some kind of spiritual interference (for this distinction see Bourguignon 1976; and Cardeña et al. 2009). This distinction is sometimes difficult to make, as evidenced in the study of Sapkota et al. (2014), in which medically unexplained fainting spells were attributed to spirit possession rather than necessarily involving episodes of a replacement of the usual identity for another one.

The experience of pathological possession is now included in the DSM-5 under the description of identity disruption, a feature of Dissociative Identity Disorder (DID), on the condition that the alterations in consciousness are distressing and/or dysfunctional, and that the disturbance is not part of a broadly accepted cultural or religious practise (American Psychiatric Association 2013:292). However, empirical evidence for the validity of possession states as a psychopathological phenomenon and its possible connection to trauma is still scarce (Neuner et al. 2012:549), and Van Duijl et al. (2010) have recommended more quantitative research on this link.

### Spirit Possession, Dissociation, and Trauma

Spirit possession has been etically discussed under the umbrella of dissociative psychological processes (e.g., Cardeña 1992; Spiegel et al. 2011). Dissociation is a complex construct involving experiential detachment such as depersonalization, and/or cognitive and phenomenal lack of access to psychological processes that are ordinarily accessible, as in psychogenic amnesia or the experience of alternate identities (Cardeña 1994; Cardeña and Carlson 2011). Western psychology and psychiatry have defined possession as dissociative because it can be described as the “splitting” of the experiencing ego into two or more identities (cf. Janet 1919/1976), and Wimmer (1924/1993) specifically discussed cases of unwilled possession from a dissociation perspective. Dissociative phenomena are not necessarily pathological and may be part of a culturally sanctioned practice associated with creative activities, a religious meaning system, or manifest as innocuous spontaneous fluctuations in ordinary conscious experience (Cardeña 1997; Maraldi 2014). Yet, dissociative reactions can also constitute a response to stress or trauma (Dalenberg et al. 2012; Hinton and Lewis-Fernández 2010; Lewis-Fernández et al. 2007; Nijenhuis 2004; Spiegel et al. 2011; Van Ommeren et al. 2001) that may become dysfunctional and bring about acute or long-term psychopathology (Cardeña and Carlson 2011).

The notion that dissociative symptoms develop in response to traumatic events was already present in Freud’s, Breuer’s and Janet’s scientific works on hysteria, and in Prince’s scholarly pieces on multiple personality and dissociation (Spiegel and Cardeña 1991). Because a considerable research database, mostly but not exclusively from industrialized nations, has shown a clear link between traumatic events and acute and long-term dissociative manifestations (Dalenberg et al. 2012), various studies have discussed possession as a dissociative phenomenon perhaps triggered by traumatic experiences (Igreja et al. 2010; Neuner et al. 2012; Van Duijl et al. 2010). For instance, systematic observation in Sudan revealed that (*zar*) possessed females had typically undergone pharaonic circumcision (female genital mutilation) (Boddy 1989), within a culture described as a “culture of pain” (Boddy 1998:104). In Uganda, health workers and counselors, but not traditional healers, discussed a link between trauma and possession, and all three groups saw a relation between trauma and dissociative amnesia or depersonalization (Van Duijl, Cardeña, and de Jong 2005). In another study, some of the same authors (Van Duijl et al. 2010) found trauma to be a general predictor of dissociative phenomena, including possession. In their study, possessed individuals reported more cumulative potentially traumatizing events, as well as more events related to health, injury, and having been in mortal danger than a non-possessed comparison group, but attributed possession to socio-cultural factors rather than traumatic events. Furthermore, the possessed respondents also scored higher on somatoform dissociation. Also in Uganda, Neuner et al. (2012) reported that extreme levels of trauma, such as those found among abducted child soldiers, were associated with high levels of dissociation including possession phenomena. In a study in a low SES, conservative part of Turkey, Sar, Alioğlu, and Akyüz (2014) found that spirit possession and various types of ostensible psi phenomena such as telepathy were reported across their sample, but were substantially more prevalent within the most traumatized group who also exhibited dissociative and trauma-related disorders.

In American countries, the most commonly studied cultural expression of distress may be *ataque de nervios* (literally: attack of nerves) (e.g., López et al. 2011), but possession is also common particularly in some countries (Brazil, Cuba, the Dominican Republic, Haiti, and Venezuela). Spirit possession has been interpreted as corporeal display of recent collective violence and distress in Venezuela (Ferrándiz 2009) or a form of social memory in the aftermath of slavery in Cuba (Pichler 2010). Hitherto, quantitative research on possession/mediumship (mediumship is a broader term than possession, but sometimes is used synonymously with it) and dissociation has been undertaken, to the best of our knowledge, only in Brazil and Cuba. In research with 110 mediums from a Kardecist Centre in São Paulo, Negro, Palladino-Negro, and Louzã (2002) found that controlled dissociation was positively related to formal training in mediumship, with mediums reporting good socialization and adaptation. Dysfunctional forms of dissociation were associated with younger age, less control of mediumship activity, poor social support, and previous psychiatric history. Seligman (2005a) compared Candomblé mediums with nonmedium initiates, frequenters of spiritual centres, SES-matched controls, and high-SES controls. She proposed that a tendency to dissociate is not the defining characteristic of Candomblé mediumship because other Candomblé practitioners had similar dissociation scores, although they had significantly higher scores than the two comparison groups. She concluded that somatization is a cardinal aspect differentiating mediums from other groups, but reported that Candomblé mediums exhibited only “a trend” towards greater somatization than the other comparison groups (after removing high-SES controls and thus poverty as potential cause for somatization). Because of the small size of her sample (only 11 initiated mediums) and lack of clarity in her report as to whether the mediums differed significantly from the other Candomblé groups, her conclusions must be taken guardedly.

Also in Brazil but with a different group (Kardecist Spiritists) Moreira-Almeida, Lotufo Neto, and Greyson (2007) reported that their sample of 115 mediums’ social adjustment was comparable to that of the general population and better than that of psychiatric patients, and the mediums had a lower prevalence of common mental disorders than that observed in other studies with non-clinical groups. Moreira-Almeida, Lotufo Neto, and Cardeña (2008) compared data of a sample of Brazilian mediums with those from dissociative disorder identity patients from other countries. The first group reported less history of trauma and better indexes of psychological health and social adjustment than the latter. Similarly, in Cuba Laria (1998) compared three groups: spirit mediums, mental health patients, and a comparison group. Mediums reported higher levels of “normal” dissociative experiences, lower levels of psychopathology, fewer traumatic experiences, and less subjective distress than mental health patients, although they had experienced more stressful events than the comparison group.

### Spirit Possession and Local Context

Located in the Eastern part of the Caribbean isle of Hispaniola, and previously colonized mostly by Spain, the Dominican Republic is a predominantly Catholic country with an increasing number of protestant evangelical congregations (CIA 2014; U.S. Department of State 2005). The country, however, has a strong African heritage and has preserved some aspects of African religions and evolved them into what nowadays is called *21 divisiones* or *brujería*, a Dominican variety of Haitian *Vodou*. An important difference between the two varieties is that Dominican *Vodou* is less structured but more influenced by European Christian and kardecistic elements. The practice of *Vodou* involves singing and dancing, elaborate dress, ritual practice, and connection to the spirits (Davis 1987; Deive 1975/1996).

The Dominican Republic differs from some other countries in which possession has been researched after their recent history of enormous social violence and upheaval. Although the Dominican Republic is not free from poverty and social problems, it has not undergone mass violence for a number of decades. Previously published studies that relate spirit possession to traumatic experience, in contrast, have generally been conducted in areas haunted by collective violence, such as Uganda (e.g., Van Duijl et al. 2010) or Mozambique (Igreja et al. 2010). An important research question is whether a link between trauma and the practice of spirit possession can be observed in non war-ravaged countries that nonetheless experience considerable everyday violence, such as some Latin American and Caribbean countries.

*Vodou* in the Dominican Republic differs from the practices of “central morality” of the elites, and may be seen as an alternative of the powerless against the powerful (Lewis 1971/1989:127). Poor women generally rank among the most disenfranchised groups and predominate as possession practitioners throughout the globe (e.g., Boddy 1989; Schmidt 2010). Women may gain in status as they are inducted into membership of the possession cult group and graduate into the position of a female healer. Furthermore, they may gain in self-control as “what is considered to begin with as an uncontrolled, unsolicited, involuntary possession illness” readily develops “into an increasingly controlled, and voluntary religious exercise” (Lewis 1989/1971:83).

The specific characteristics of *Vodou* are not easy to classify. Its spirits, in the Dominican Republic commonly referred to as *misterios* (mysteries), vary from benign to aggressive. Once they arrive in the ritual space they “mount” (possess) their (human) horses, who are said to be “ridden”, a metaphor used cross-culturally, for instance in rural Nepal (Sapkota et al. 2014). Whether or not the spirits harm their human hosts is said by some practitioners to depend on the ability of the latter to master possession. Although some authors describe *Vodou* possession in general as a culturally sanctioned benevolent variety of possession, a privilege only available to a chosen few who possess certain mental predispositions (see Bourguignon 1976; Davis 1987; Desmangles and Cardeña 1994), it has also been stated that *Vodou* possession often follows affliction (Agosto Muñoz 1972; Lewis 1971/1989:60).

General experiences at the onset of and during possession by the *misterio* spirits include unusual sensations in different body parts; emotional alterations; changes in voice and face expression, posture and gesture, vision and hearing; as well as the experience that some entity has taken control over one’s body, which results in a changed sense of identity (Schaffler et al. in preparation). The spirits are invoked and worshipped with their corresponding songs, ritual prayers, gestures, salutations, and drawings on the ground with flour (*firmas or veves*), as well as with offerings that consist of different types of food, sodas, and alcohol (Davis 1987). The most important cues to trigger possession are drums and dance (Rouget 1985). However, the spirits also manifest spontaneously, within and outside the ritual setting, for instance with the intention to deliver urgent messages. Unbidden possession may also have violent features. In general, though, practitioners seek to transform unintentional possessions into intentional, “executive” ones (cf. Sapkota et al. 2014).

The first author conducted extensive fieldwork in the Dominican Republic, (Schaffler 2009a, b, 2012, 2013, 2015) and observed that *vodouists*, especially at the beginning of their careers, suffer at times from violent possessions as they flail about or convulse before falling to the ground where they are tossed about. This phenomenon is emically referred to as *caballo lobo* (literally: wolf horse) in the Dominican Republic, or *chwal gate* (literally: spoiled horse) or *bossal* possession in Haiti (Bourguignon 1965:49). Aside from acute states of hard to control possession with violent features, spiritual development is reported to be accompanied by headaches, stomach-aches, vertigo, or other somatic symptoms (Schaffler 2009a, 2012, 2013).

Concerning the phenomenal aspects of possession, experience is of vital importance. Generally speaking, the more experienced possessed individuals are, the more their possessed actions are controlled and follow a cultural script. Readily initiated individuals are expected to carry out symbolic acts during public celebrations (*fiestas*) involving singing and drumming, or during private counselling sessions (*consultas*), with the intention to influence positively the fate of their peers (Bourguignon 1976:40).

In the present study, we inquired about two common experiences of possessed individuals, namely about not being able to control the moment when possession starts (unbidden possession), and about the spirits entering them in such gross fashion that they fall down and are tossed about (violent possession). Unbidden possession is characterised by a lack of control of the moment of possession. Spontaneous communications through the spirits are usually considered valuable, and being contacted by the spirits without consent is seen as a sign of spiritual election rather than of illness. Violent possession, on the other hand, is both unbidden and renders the individual unable to speak or perform ritual actions. This phenomenon, which in extreme cases may resemble epileptic seizures, is emically explained as the spirits entering with too much force the body of an inexperienced and thus “spiritually weak” individual (*caballo lobo*), or interpreted as punishment by the spirits (*castigo*) for neglected spiritual duties (Schaffler 2009b:121ff, 2015). Because illness related to spirit possession may cause much affliction, some *Vodou* centres offer the possibility of temporary hospitalization (Schaffler 2009b:273ff). Local treatment consists in rituals that aim at strengthening the affected individual’s spiritual force or at reconciliation with the angry spirits. Affected individuals may also seek medical treatment.

After experiencing possession, individuals usually report amnesia for the possession, but authors have described a variety of alterations of consciousness, not all of them followed by amnesia (Bourguignon 1976:40; Cardeña 1989, 1992; Frigerio 1989; Van Duijl, Kleijn, and de Jong 2013). In Dominican *Vodou*, one specific level is described as a state of slight consciousness alteration during which the *misterio* spirits only “touch” or “inspire” people without actually possessing them. This level is, however, emically considered to be different from possession. It is seen as less dangerous in that it never leads to loss of control, and non-possessed individuals may experience it, too. Such state of inspiration is a prerequisite for *vista clara* (literally: clear sight), the ability to use spiritual inspiration for the purpose of foretelling (for a similar division among Cuban practitioners, see Espirito Santo 2014). The *misterios* are assumed to inspire their devotees’ thoughts and dreams or send them signs in the environment, for instance through manipulating the shape of clouds, ashes, watermarks, or candle wax. Like unbidden possession, these communications through the spirits are interpreted as signs of spiritual election and we included them as possible predictors of possession.

### Study Objectives

Our cross-sectional study evaluates possible predictors of *Vodou* possession in the Dominican Republic, on which there has been little research so far. Using a comparison of individuals who do or do not experience possession, we examined the association of spirit possession with the experience of traumatizing events, somatoform dissociation, and problems with sleep. We predicted that those who experienced possession would differ significantly from SES-matched non-possessed peers in terms of environmental and familial influences because we believed that dissociative behaviour could be either socially learned or an inherited dissociative tendency. We predicted that spirit possession would be related to experiential predictors such as dreams, visions or unbidden thoughts attributed to the spirits, and to friends or family having reported that an individual would some day become possessed.

Since narratives about *Vodouist* spiritual development often refer to the experience of unbidden and violent possession, we inquired about the frequency of such experience, assessed the possessed individuals’ degree of suffering due to violent possession, and hypothesized that a segment of the possessed group had already seen a physician for violent possession and symptoms they connected to spirit possession.

## Methods

### Study Background

This study is part of a long-term research project, conducted by the first author among Dominican *vodouists*. The project’s overall methodological approach is based on Grounded Theory (Glaser and Strauss 2005), in which observations from early stages serve to ground later hypotheses in a self-enriching and self-correcting circle. It comprises narrative interviews focusing on biography and spiritual development, and problem-centred interviews including a range of questions regarding the religious practice itself. Other methods included participant observation and videography of rituals of possession.^1^ The hypotheses tested in this paper were partly derived from the analysis of the qualitative data and from a review of the literature.

The practice of spirit possession is mostly found in the Southwest of the Dominican Republic, commonly referred to as *El Sur*. The study was conducted in urban and suburban venues of the South, particularly in areas in and around the cities of Santo Domingo, San Cristóbal, and Baní, characterized by poverty, infrastructural problems, and an absence of tourism. Especially in the Dominican cities of the South, violence conducted with weapons and armed robberies are a serious and increasing problem. Among the primary factors contributing to this increase are large scale migration to urban areas, unemployment, domestic violence, abuse of drugs and alcohol, drug trafficking, and the availability of weapons (OSAC 2013). Moreover, violence against women including femicide is high (CLADEM 2008; ONE 2009).

Because of its lack of formal structure, the actual size of the Dominican *Vodou* community is hard to estimate. According to current practitioners, decades ago it was difficult to find a spiritual centre where possession was carried out as openly as it is today. Back then, it was first prohibited (Davis 1987:40f) and then seen as a practice of Haitian immigrants or Dominicans who inhabited the distant countryside or the former centres of sugar production (e.g., Bogaert García 1992:182). Today, spiritual centres are frequently situated in poor suburban neighbourhoods of the South. It is “hard to find anybody here who is not either a devotee of *Vodou* or a Pentecostal church,” as one possessed individual put it to the first author. It has to be highlighted though, that *Vodou* is not the dominant religious system in the Dominican Republic. It thus frequently occurs that advocates of a “pure” Christian religion pressure *vodouists* to give up their beliefs, disavowing their spirits as demons, and, as a result, many *vodouists* hide their beliefs from others. Also, there is a high fluctuation between religious systems due to the high-pressure evangelization strategies by Protestant evangelical congregations such as Pentecostals, Adventists, and Mormons. In the course of the last decades, the traditional socio-demographic characteristics of possessed individuals have been changing. A variety of social conditions contemporaneous with patterns of urbanization and modernization have contributed to the decline of females in Dominican popular religion (Piper 2012). Furthermore, Dominican *Vodou* has become increasingly attractive for homo- or transsexual men who enjoy having their male bodies invaded by female spirits, a phenomenon also observed in Brazilian Candomblé (Birman 1995; Van der Port 2005). As in Haiti, male homosexuals are seen to be under the protection of certain feminine spirits, allowing them to exhibit stereotypical feminine behaviours during religious ceremonies, and an emic explanation is that these female spirits caused their sexual orientation (Lescot and Magloire 2002). According to the first author’s observations, spiritual centres owned by a male homosexual serve as a meeting point for other male homosexual or transsexual possessed individuals who thus express their sexual orientation relatively openly. Even among *vodouists*, these centres are only partly accepted, and elder members of the community may criticize this development because it contributes to the public reputation of the cult as “immoral.”

In general, Dominican *vodouists* tend to be dark-skinned and marginal to the formal economy in that they are either unemployed or work informally as vendors, janitors, or maids for the wealthier citizens. They not only face racial discrimination (Candelario 2007) but also the problems that come along with societal segmentation, such as being confronted with an inaccessible wealthier lifestyle. Women additionally are seen as subservient to men, are left alone with domestic work (ONE, UNICEF, OIT 2011:45), and have a lower earned income than men. In a patriarchal organized society that is rather conservative when it comes to gender roles, homosexual males are likely suffer from additional discrimination.

### Sample Selection and Procedure

We conducted a questionnaire- and interview-based survey among 85 citizens of Dominican or Dominico-Haitian descent, comparing 47 individuals that had experienced possession with 38 who also follow *Vodou* but had not experienced it. Both possessed and non-possessed participants were *vodouists* who regularly visited spiritual centres and participated in their celebrations. Possessed individuals either owned spiritual centres or joined them, for instance to manifest the *misterio* spirits, keep up friendships, or, if they struggled with unbidden or violent spirit possession, to seek advice from their more experienced peers. Non-possessed individuals visited spiritual centres primarily to enjoy direct interaction with the spirits that manifested in the bodies of those possessed, and in order to improve their health, business, or love life. This means that they were most often the clients of possessed individuals. Further motives for non-possessed individuals to visit spirituals centres included exerting non-possessed ritual functions such as being a ritual assistant (*plaza*), a litanist, a singer or a drummer, or being with friends.

Study participants were approached either at spiritual centres or at stores (*botánicas*) selling *Vodou* paraphernalia. Since *vodouists* are sometimes exposed to persecution by fundamentalist Christians and thus tend to hide their practice, we depended on already well-known experienced practitioners. The latter served as key informants who identified and introduced us to owners of spiritual centres or stores, where we requested visitors to participate in our study. Due to the high illiteracy rate in the study area, the first author interviewed the participants face to face in the local language (Spanish) in January and February 2013.

At the beginning of the interview, participants were asked to indicate whether or not they had experienced full possession by the *misterio* spirits at least once in their lifetime. Those who answered the question “yes” were classified as possessed; those who answered “no” were classified as non-possessed individuals. Pilot testing with 21 possessed individuals showed that whereas some items in the Spanish version of the Somatoform Dissociation Questionnaire (SDQ-20) (Nijenhuis et al. 1996) were not easily understood, all items of the SDQ-5 were comprehensible. The entire ad-hoc questionnaire, phrased to follow the local language, was easily understood. All study participants gave verbal consent and procedures followed the Helsinki declaration. The study was approved by the Ethics commission of the Medical University of Vienna.

### Questionnaire Instruments

#### SDQ-5

The *SDQ*-*5* is a 5-item instrument of very common somatoform symptoms, with a scale ranging from 1 = never to 5 = almost always (Nijenhuis et al. 1997). These symptoms have been strongly associated with exposure to potentially traumatizing events including physical threats (Nijenhuis 2010). At least in a Western context, these 5 items discriminate best between patients with dissociative disorders from those without these disorders (Nijenhuis et al. 1997). Sensitivity, specificity, positive predictive value, and prevalence-corrected negative predictive value range from satisfactory to excellent. A score of ≥8 is likely to predict a dissociative disorder. Even though the SDQ-5 was more sensitive than the DES to assess dissociative pathology among patients with somatoform disorders among Dutch psychiatric patients (Nijenhuis 2010), it performed less well in a sample of Turkish psychiatric patients (Sar et al. 2001). We used the SDQ-5 items found in the Spanish version of the SDQ-20 (Nijenhuis, van der Hart, and Vanderlinden et al. 2002a). In our sample, the internal reliability of this scale was moderate (α = 0.66), which is not that low considering the few items in the scale. Because dissociative states have been associated with sleep disturbances (American Psychiatric Association 2013), we added a question regarding the quality of sleep (“Do you sleep well at night?”). To find out whether possession is generally associated with urogenital problems or if this applies only to societies where clitoredectomy is performed (Boddy 1989), we added a second question derived from the SDQ-20 (“Do you feel pain in your genitals at times other than intercourse?”). As recommended for the SDQ-20 and the SDQ-5, our study participants were asked after each item to answer yes or no to the question, “Is the physical cause known?” The authors of the questionnaire recommend not adjusting the scores when the participants report a known cause of a physical symptom (Nijenhuis, van der Hart, and Vanderlinden et al. 2002a), and we followed their suggestion.

#### TEC-DR

A 7-item ad-hoc version of the *Traumatic Experience* Checklist (TEC-DR) (Nijenhuis, Van der Hart, and Kruger 2002b) measured the perceived effect of potentially traumatic experiences, according to a 5-point scale ranging from 1 = no influence to 5 = very strong influence. We selected seven items relevant to our research hypothesis out of the 33-item original TEC (“When you were a child, did you have to work to make a living or take care of your siblings and thus took a lot of responsibility?”, “When you were a child, were you left by one of the persons who brought you up or did one of them die?”, “Did you experience a mortal danger like an assault, an accident or a disease?”, “Did you observe a serious accident/an act of violence that was done to another person?”, “Did you experience physical violence like being hit, being slapped, being shoved, being whipped?”, “Did you experience sexual harassment or other forms of sexual abuse?)”, and “Have you been violated?”. In our sample, the internal reliability of this scale was moderate (α = 0.62).

#### SPQ-DR

We used the ad-hoc instrument *Spirit Possession Questionnaire*-*Dominican Republic* (*SPQ*-*DR*) to collect data on demographic and predictors of possession (e.g., participants’ family background, environmental and familial influences). The *SPQ*-*DR* items investigated possible influences of social environment/family with a 5-point scale ranging from 1 = no influence to 5 = very strong influence. The internal reliability of this scale was good (α = 0.82).

The *SPQ*-*DR* also included seven questions regarding spirit possession that we solely asked to possessed individuals. We assessed frequency of involuntary or violent possession with two items (“Do the spirits still mount you without invocation?”, “Do they continue to drag you around or throw you on the floor?”) using a 5-point Likert Scale ranging from 1 = never, 2 = sometimes/year, 3 = sometimes/month, 4 = sometimes/week, 5 = sometimes/day. Both items were accompanied by a multiple answer question assessing the setting in which these incidents happen, offering three answer choices: “in a ritual context”, “in private with family or friends”, and “in public”. Also, we explored the current level of suffering that study volunteers experienced due to violent possession with the question “Do you suffer because they continue to drag you around and throw you on the floor?” using a 5-point Likert Scale ranging from 1 = none to 5 = very much. Further items were “Have you seen a doctor/psychiatrist because of spiritual affliction, that is being ridden spontaneously, being thrown on the floor, or suffering inexplicable pain?” and “Was the doctor/psychiatrist able to help you?”. Given that among *vodouists* admitting that they remember what they said or did during possession suggests that they have not been really possessed (Métraux 1955:33), we decided not to ask about the occurrence of amnesia to avoid implicitly challenging participants and just probably receiving socially desirable answers.

### Analysis

Single missing values with random distribution were tolerated without interpolation approaches to avoid loss of power. We investigated subgroup differences using *χ*^2^ (chi sq) tests for categorical variables, *t* tests for interval variables, and Mann–Whitney’s *U* test for interval variables with non-normal distributions. For variables in which subgroups differed significantly, we performed a discriminant function analysis to assess the extent to which these variables discriminated between subgroups. For this analysis, we set the cut-off for interpretation of structure matrix loadings at 0.3 and used Wilks’ lambda statistic for testing the significance of the model. To test the intercorrelations across questions, we conducted a Principal Component Analysis (PCA) with varimax rotation and used as cutoff a variable loading of 0.4. Data of completed questionnaires was statistically processed using SPSS version 17.0. Statistical significance was set at *p* < 0.05, two-tailed.

## Results

### Demographic Characteristics

None of the questionnaires was considered unreliable due to having more than 5 % missing data, thus all questionnaires were included in the data analyses. Table 1 depicts demographic characteristics. Only adults were eligible for participation in this survey and the sample consisted of 85 adult participants (possessed, henceforth referred to as P = 47, non-possessed, henceforth referred to as NP = 38) with a similar gender distribution, particularly among the P. The majority of participants reported being in a relationship, having completed primary education or less, having been born in an urban setting and still living in such a setting, and never having been a member of a Protestant church. With regard to SES, majorities of both groups considered themselves to be “the same” or better than their peers (84 % of P versus 76 % of NP). The only demographic variable in which the groups differed significantly was profession, in which 36 % of P identified themselves as spiritual counselors as compared with 3 % of the NP.

**Table 1:** Sociodemographic characteristics

Factors	Groups		Total
	Possessed	Non-possessed	
	*n* (%)	*n* (%)	*n* (%)
Total	47 (55)	38 (45)	85 (100)
Males	23 (49)	15 (40)	38 (45)
Females	24 (51)	23 (60)	47 (55)
Education			
Dropout (<8 years)	20 (43)	12 (32)	32 (38)
Primary (8 years)	13 (28)	13 (34)	26 (31)
Secondary (4 years)	13 (28)	9 (24)	22 (26)
University (4 years)	1 (2)	4 (10)	5 (6)
Duration (in years)	8.3 (4)	9.5 (4)	8.9 (4)
Profession*			
Day-worker	12 (25)	15 (40)	27 (32)
Regular worker	4 (8)	9 (24)	13 (15)
Spiritual counsellor	17 (36)	1 (3)	18 (21)
Non-worker	14 (30)	13 (34)	27 (32)
SES			
Much worse	1 (2)	1 (3)	2 (2)
Worse	6 (13)	8 (22)	14 (17)
Same	26 (55)	20 (54)	46 (55)
Better	11 (23)	7 (19)	18 (21)
Much better	3 (6)	1 (3)	3 (4)
Birth place			
Rural	18 (38.3)	19 (50.0)	37 (43.5)
Urban	29 (61.7)	19 (50.0)	48 (56.5)
Residence			
Rural	6 (12.8)	6 (15.8)	12 (14.1)
Urban	41 (87.2)	32 (84.2)	73 (85.9)
In a relationship			
No	18 (41.9)	19 (51.4)	73 (46.2)
Yes	25 (58.1)	18 (48.6)	43 (53.8)
Ex-member of non-Catholic church			
No	31 (66.0)	28 (73.7)	59 (69.4)
Yes	16 (34.0)	10 (26.3)	26 (30.6)

Factors	Groups		Total
	Possessed	Non-possessed	
	*n* (%)	*n* (%)	*n* (%)
	Mean (SD)	Mean (SD)	Mean (SD)
Total age	37.5 (11.8)	33.9 (11)	35.9 (11.6)
Age males (years)	34.1 (11.1)	29.8 (6)	–
Age females (years)	36.4 (13.0)	36.6 (13.0)	–

* *p* < 0.05

### Predictors of Possession

With respect to the degree of environmental and familial influences there were no significant differences on how many family members from the household practiced spirit possession (*M* = 1.91, *SD* = 1.16), or how many other family members not in the household practiced possession (*M* = 2.78, *SD* = 1.77). Regarding previous traumatic events, we found a marginal difference (*p* = 0.07) as the P group reported more frequent experiences of mortal danger such as assaults, accidents or diseases, than the NP.

With regard to the questions about experiential predictors of possession (Table 2), P reported overall more signs emically associated with possession but differed significantly only in one item (individuals interpreting more signs in the environment as coming from spiritual beings), and differed marginally in another (unbidden thoughts attributed to the spirits, *p* = 0.06). The groups did not differ in having more dreams in which spirits appeared or in friends or family reporting that they would some day become possessed.

**Table 2:** Predictors of possession

Question	Groups mean (SD)		Effect size (η^2^)
	Possessed (*n* = 47)	Non-possessed (*n* = 38)	
(1) Did friends or family members predict that you were going to be possessed one day?			
	2.2 (1.6)	1.7 (1.3)	0.025
(2) Did you have dreams in which the spirits appeared?			
	2.9 (1.6)	2.4 (1.5)	0.021
(3) Did you see signs in the environment you thought indicated the presence of the spirits?			
	3.1 (1.7)	2.1 (1.6)	0.095**
(4) Did you have unbidden thoughts you thought were messages from the spirits?			
	3.1 (1.8)	2.4 (1.7)	0.040
SC total score			
	2.9 (1.3)	2.1 (1.2)	0.081**

** *p* ≤ 0.01

**Fig. 1:**
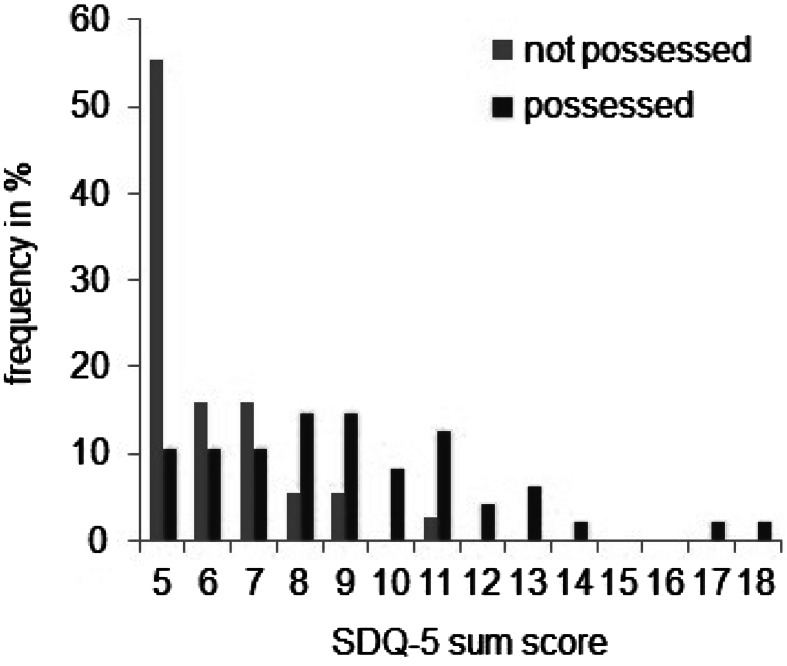
Scores in the SDQ-5 by group

As depicted in Table 3, we found clear differences between P and NP concerning somatoform dissociation and sleep. Besides the overall difference in the SDQ scale, the P had higher scores in the following items: having the body or parts of it being insensitive to pain, seeing things around differently than usual, feeling that the body or parts of it have disappeared, being unable to speak or speaking only in a whisper, and not sleeping well at night. The groups did not differ in trouble urinating or feeling pain in the genitals. Taking into account only questions from the original SDQ-5 and using the proposed cut-off of ≤8 for caseness, 68 % of the P would qualify as cases compared with 13 % of the NP, with the former group also having the most extreme scores in the SDQ (see Fig. 1).

**Table 3:** The SDQ-5 + sleeping question and genital question

Question	Groups mean (SD)		Effect size (η^2^)
	Possessed (*n* = 47)	Non-possessed (*n* = 38)	
Do you have trouble urinating?			
	1.0 (0.1)	1.1 (0.3)	0.043
Is your body or a part of it insensitive to pain?			
	2.0 (1.1)	1.1 (0.4)	0.255***
Do you see things around you differently than usual?			
	2.1 (1.1)	1.1 (0.4)	0.242***
Do you feel as if your body or a part of it has disappeared?			
	2.0 (1.0)	1.3 (0.6)	0.153***
Does it happen that you cannot speak or only whisper?			
	2.0 (1.1)	1.5 (0.9)	0.054*
SDQ scale			
	1.8 (0.6)	1.2 (0.3)	0.004***
Do you sleep well at night?			
	3.6 (1.3)	4.3 (0.9)	0.082*
Do you feel pain in your genitals (at times other than intercourse)?			
	1.1 (0.5)	1.1 (0.3)	0.000

* *p* ≤ 0.05; ** *p* ≤ 0.01; *** *p* ≤ 0.001

We conducted a discriminant analysis to evaluate which variables predicted assignment to P or NP. Two scales (SDQ-5 and SPQ-DR) and two questions (the sleeping question and the trauma item “Did you experience a deathly experience like an assault, an accident or a disease?”) differentiated the two groups and were used as discriminant factors. In total, 79 valid cases could be used in the analysis for P and NP individuals. Initially all predictors were entered simultaneously. Based on the structure matrix, four predictor variables were associated with the respective discriminant function (−0.796 versus 0.738), which distinguished between the two groups: the original SDQ-5 scale (*r* = 0.93), the sleeping question (*r* = −0.46), the SPQ-DR scale (*r* = 0.40), and the question concerning the experience of mortal danger (*r* = 0.30). Next, we entered the predictors stepwise to reveal items with the best predictive potential for group classification. The SDQ-5 was the best predictor variable (Wilks lambda = 0.656, *χ*^2^ = 32.3, *p* = 0.0001). Table 4 shows that the cross-validated accuracy rate was 72 %, which was greater than the proportional by chance accuracy criteria of 63 % (1.25 × 50.1 %), with 74 % of the original cases being correctly accounted for by the predictors.

**Table 4:** Discriminant analysis

Group membership	Predicted group membership		Total	Correct prediction
	Possessed	Non-possessed		
	*n* (%)	*n* (%)	*n* (%)	%
Original				
Possessed	31 (66.0)	16 (34.0)	47 (100)	
Non-possessed	6 (15.8)	32 (84.2)	38 (100)	74.1
Cross-validated				
Possessed	30 (63.8)	17 (36.2)	47 (100)	
Non-possessed	7 (18.4)	31 (81.6)	38 (100)	71.8

We also conducted a factor analysis to evaluate the associations across variables. Our initial analysis on all the questionnaire items gave a six-factor solution, which included single variable factors, so we opted for a 3 factors solution, which accounted for 46 % of the variance (Table 5). The first factor can be thought of as a being responsible/taking on a professional spiritual role and included: taking a lot of responsibility as a child, having friends or family members predict that the person would be possessed, having dreams in which the spirits appeared, seeing signs in the environment indicative of the presence of the spirits, and having thoughts attributed to the spirits. The second factor is a trauma/pain factor with the following variables: experiencing mortal danger, having been exposed to sexual abuse and violation, and having genital pain. The final factor is a well-being/low dissociation factor and includes sleeping well and low levels of the following: insensitivity to pain, seeing things differently than usual, feeling the body disappear, and being unable to speak.

**Table 5:** Factor analysis of questionnaire items (varimax rotation)

Variables	Factors		
	1	2	3
Responsibilities in childhood	0.567		
Friends or family predicting	0.782		
Dreams with spirits	0.731		
Signs in the environment	0.761		
Unbidden thoughts as messages	0.719		
Mortal danger		0.490	
Physical violence		0.563	
Sexual harassment/abuse		0.683	
Violation		0.788	
Pain in genitals		0.654	
Body insensitive to pain			0.653
Seeing things differently than usual			0.723
Body or parts disappeared			0.703
Cannot speak			0.695
Sleeps well			−0.454
Eigenvalue	3.03	2.68	2.63
Percent of variance explained	16.83	14.89	14.68

### Features of Spirit Possession

In the additional items only asked to P, 43 % of them reported unbidden possession “several times a year”, 21 % “several times a month”, and 9 % “several times a week” or more frequently; 66 % reported unbidden possession in a ritual setting, 38 % in a private setting with family or friends, and 15 % in public settings. With regard to violent possession, 32 % of P reported to being dragged around and thrown on the floor “several times a year,” 11 % underwent violent possession “several times a month” or with a higher frequency; 34 % reported that violent possession occurred in a ritual setting, 15 % in private with family or friends, and only 2 % in public settings. With regard to current level of suffering caused by possession, 17 % described it as “mild” or “moderate,” whereas 2 % admitted to suffering “quite a bit”. At some point in their lifetimes, 34 % of P had sought medical advice due to symptoms such as being ridden spontaneously, being thrown on the floor or suffering inexplicable pain, without getting satisfactory treatment.

## Discussion

### Demographic Characteristics

The two groups did not differ demographically except that the P seemed to have developed a socioeconomic advantage from their experiences by using it professionally. With reference to gender differences, we did not replicate the greater incidence of women than men among possessed individuals reported by other authors (e.g., Boddy 1989; Schmidt 2010), perhaps as a result of a change in social conditions that has caused a shift in the gender ratio and because emerging non-traditional gender identities have produced a recent influx of homosexual and transsexual males (Piper 2012). To these males, possession could appeal for the same reasons as for women, as it may help advance their interests and improve their lot (Lewis 1989/1971:127).

### Predictors of Possession

With respect to previous traumatic events, the P group reported more frequent experiences of mortal danger such as assaults, accidents or diseases, than the NP, but the difference was only marginal. A previous history of trauma was thus not a clear predictor of possession.

Regarding experiential predictors of possession, P overall reported more of them but the only item that was independently significant was that they saw more signs of spiritual forces in the environment. A tendency to give meaning to patterns was also found in a psychoanalytic study in which P and NP females from the Dominican Republic were tested with the Rorschach projective technique. The P group was more interested in details and saw more human details in the inkblots, from which the author concluded that they processed information in a more creative way (Bogaert García 2000), as did traditional Zinacanteco shamans (Shweder 1972). Because in *Vodou* (and shamanism in general) seeing signs is interpreted as a positive form of spiritual contact, it is reasonable to us that P might experience them in their daily lives without finding them dysfunctional or disruptive. In fact, spending time engaged in reverie or other forms of absorption may be adaptive in everyday life (Cardeña, Lynn, and Krippner 2014; Seligman and Kirmayer 2008). Furthermore, since both groups often endorsed prophetic dreams, visions, and unbidden thoughts attributed to the spirits, we may conclude with Seligman (2005a), that mere alterations in consciousness are not the central and defining predictor of possession.

As far as somatoform dissociation, P endorsed more items except for problems when urinating, which had a very low incidence in our sample. Thus, the widespread urogenital problems found in the *zar* cult (Boddy 1989), which may combine long-term physiological damage with somatoform reactions, cannot be generalized to Dominican Republic women, who are not exposed to clitoridectomy or similar practices.

In our study, somatoform dissociation was the strongest variable differentiating P from NP. These results might support the somatization theory of spirit possession, according to which somatic susceptibilities cause certain individuals to identify with the possessed role and predispose them to dissociate (Seligman 2005a).

However, an alternative explanation is that some of the phenomena in the somatoform dissociation questionnaire (e.g., feeling that the body or part of it had disappeared) could refer to experiences of (ritual) possession, rather than to chronic symptoms outside of that context. Whether the P group had more manifestations of somatoform dissociation *previous* to and *outside* of the possession context should be investigated in a study that clearly evaluates a longitudinal history of the phenomena and in which context they ensue. Nonetheless, we should mention that P usually negate that they can remember what happened during the possession episode. Accordingly, their answers should in theory refer to symptoms outside the possession context, but there is reason to believe that the postulated amnesia, at least in part, corresponds more to their cosmological idea than to their actual experiences, which may vary in intensity and the presence of amnesia. Furthermore, chronic somatic symptoms previous to and outside the ritual context may be interpreted as prodromal signs of possession (Van Duijl, Kleijn, and de Jong 2013) and possibly change with progress in training. Moreover, these symptoms may be both seen as normal and beneficial (e.g., in that the spirits manifest themselves to warn about danger) and as causing affliction, which furthermore complicates categorization according to Western standards.

Three interesting patterns were evident in the factor analysis. The first one suggests that having many responsibilities as a child makes others see him/her as a potential spirit possession practitioner, and that the person may interpret different signs as evidence of contact with the spirits, perhaps because actual responsible adults are not present. We can wonder whether the early burden of responsibility may not result in a wish to surrender control in the possession experience. It is telling that spiritual signs did not load with either trauma or dissociation variables, although we should bear in mind that there may be a ceiling effect when dealing with a restricted sample, which could obscure a relation between these two types of variables.

The second factor referred clearly to the association among different forms of traumatizing events, including sexual ones, and genital pain. The final factor included most of the items of the SDQ-5 and the sleeping item (“Do you sleep well?”). It might be interpreted as a general dissociation/distress factor, but as mentioned above some of the items in this questionnaire may index alterations in consciousness during the (ritual) possession experience rather than pathological dissociation in general. The relation of most of the SDQ-5 items to not sleeping well may have to do with general distress/dissociation.

### Features of Spirit Possession

Whereas in Western psychiatry unintended possession has been regarded as potential evidence of psychopathology (Bourguignon 1976; Cardeña et al. 2009), we found that unbidden possession outside the ritual setting was endorsed by the majority of P, suggesting that this kind of experience is *normal* to them. On the other hand, only less than one fifth of P reported that they would currently experience violent possession outside the ritual setting or mentioned violent possession causing mild or moderate affliction. The current experience of more severe suffering due to violent possession appears to be quite rare, which is consistent with the data of Somasundaram et al. (2008) among community possession experients.

These data possibly reflect the psychosocial benefits of spirit possession such as status-enhancement and gain in self-control (Lewis 1989/1971:83), and help in developing a personal narrative that makes dissociative states controllable and organized instead of deviant and dysfunctional (Seligman 2005b). The fact that a much smaller segment of P endorsed currently experiencing affliction due to violent possession (19 %) than had sought medical advice because of illness they attributed to spirit possession (including violent possession) (34 %) in the past, suggests that suffering diminishes with increased age and/or experience, corroborating the positive relation between controlled dissociation and formal training in mediumship as found by Negro, Palladino-Negro, and Louzã (2002). This idea is supported by the claims made by possessed participants themselves, that their spiritual development started with a distressing experience of “wild” possession (*caballo lobo*) (Bourguignon 1965:49; Schaffler 2009a, 2012, 2013).

### Limitations

Regarding the instruments used in the present study, the SDQ-5 did not seem to have good diagnostic characteristics with this group. In a Western context, 43–84 % respondents with a score of ≥8 have been found to have a dissociative disorder (Nijenhuis 2010). In our sample, 68 % of P obtained a score of ≥8 (see Fig. 1). However, a much smaller segment of P (less than one-fifth) reported violent possession outside the ritual setting or current suffering as a consequence of it, and even less individuals (11 %) reported violent possession with a frequency of “several times a month” or higher. Considering that frequent uncontrolled dissociation outside the ritual setting and consequent affliction would be components of dissociative pathology, and that our factor analysis only showed a weak relation between traumatic experience and somatoform dissociation, we conclude that the SDQ-5 is not a valid indicator of dissociative disorder or traumatic experience in the local setting.

Our question concerning the quality of sleep was fruitful but too unspecific to draw detailed conclusions, so we recommend pursuing the issue with more specific questioning in future research. With regard to our questions of sexual harassment or other forms of sexual abuse, and violation there might have been some underreporting because of lack of privacy during the interview situation. The results of this study are further limited by the lack of a comparison group with no *Vodou* practice, its cross-sectional nature, and the lack of follow-up questions to clarify some of the differences seen.

## Conclusion

One of our major goals was to evaluate which variables discriminate between *Vodou* practitioners experiencing possession and those who do not. Overall, P tended to have more indicators of spiritual presence, particularly in the form of visions and (marginally) unbidden thoughts attributed to the spirits, but the difference with NP was not large. There was no significant difference in environmental or familial influences, which did not support our hypothesis that social learning or an inherited dissociative tendency would play a major role in the emergence of possession behaviour. We also tested the association of spirit possession with the experience of traumatizing events and somatoform dissociation. Our results highlight somatoform dissociation symptoms as an important factor in spirit possession, but future research should verify whether they occur independently of the possession episodes as such. However, unlike the possessed individuals in countries affected by civil war (Igreja et al. 2010; Neuner et al. 2012; Van Duijl et al. 2010), possessed vodouists in the Dominican Republic did not seem to have a remarkably more severe story of trauma than their non-possessed peers. It is also telling that in our data traumatic events correlated among themselves but did not relate to somatoform dissociation.

A secondary goal of this study was to survey the frequency and intensity of the experience of unbidden and violent possession among P, the first type of possession being characterised by a lack of control over the timing, and the second also by a lack of a ritual function, typically causing the individual to roll over the floor with flailing limbs and rendering her/him unable to speak. We found that whereas the majority of P frequently mentioned unbidden possession outside the ritual setting, the current experience of violent possession outside the ritual setting or marked affliction due to it were rare.

Our data reinforce the view that the practice of spirit possession provides economic gains (Bourguignon 1976; Lewis 1971/1989) besides other potential benefits such as developing a social network and finding meaning in and learning to control dissociative episodes. The factor relating a professional spiritual role to earlier non-traumatic events signal the need to study other personal vicissitudes and sociocultural variables in the development of spirit possession.

## Footnotes

1 All field recordings are archived at the Phonogrammarchiv of the Austrian Academy of Sciences, Vienna.

## References

[CR1] Agosto Muñoz Nelida (1972). Haitian Voodoo: Social Control of the Unconscious. Caribbean Review.

[CR2] American Psychiatric Association (2013). Diagnostic Statistical Manual of Mental Disorders.

[CR3] Birman Patrícia (1995). Fazer estilo, criando gêneros. Possessão e diferenças de gênero em terreiros de Umbanda e Candomblé no Rio de Janeiro. (Ed. UERJ).

[CR4] Boddy Janice (1988). Spirits and Selves in Northern Sudan: The Cultural Therapeutics of Possession and Trance. American Ethnologist.

[CR5] Boddy Janice (1989). Wombs and Alien Spirits: Women, Men and the Zar Cult in Northern Sudan.

[CR6] Boddy Janice (1994). Spirit Possession Revisited: Beyond Instrumentality. Annual Review of Anthropology.

[CR7] Boddy Janice, Dobash Emerson, Rusell Dobash (1998). Violence Embodied? Circumcision, Gender Politics, and Cultural Aesthetics. Rethinking Violence Against Women.

[CR8] Bogaert García Humberto (1992). Enfermedad mental, psicoterapia y cultura.

[CR9] Bogaert García Humberto (2000). Psicoanálisis de la mujer y ritos de posesión. Interamerican Journal of Psychology.

[CR101] Bourguignon, Erika 1965 The Self, the Behavioral Environment and the Theory of Spirit Possession. *In* Spiro, Melford: Context and Meaning in Cultural Anthropology, pp. 39–60. New York: The Free Press.

[CR11] Bourguignon Erika (1973). Religion, Altered States of Consciousness and Social Change.

[CR12] Bourguignon Erika (1976). Possession.

[CR13] Candelario Ginetta (2007). Black Behind the Ears: Dominican Racial Identity from Museums to Beauty Shops.

[CR14] Cardeña Etzel (1989). The Varieties of Possession Experience. Association for the Anthropological Study of Consciousness Quarterly.

[CR15] Cardeña Etzel (1992). Trance and Possession as Dissociative Disorders. Transcultural Psychiatry.

[CR16] Cardeña Etzel, Lynn Steven, Rhue Judith (1994). The Domain of Dissociation. Dissociation: Clinical and Theoretical Perspectives.

[CR17] Cardeña, Etzel 1997 The Etiologies of Dissociation. In Broken Images and Broken Selves. Susan Powers and Stanley Krippner, eds., pp. 61-87. New York: Brunner.

[CR18] Cardeña Etzel, Alvarado Carlos, Cardeña Etzel, Winkelman Michael (2011). Altered Consciousness from the Age of Enlightenment through mid 20th century. Altering Consciousness Multidisciplinary Perspectives. History, Culture, and the Humanities.

[CR19] Cardeña Etzel, Carlson Eve (2011). Acute Stress Disorder Revisited. Annual Review of Clinical Psychology.

[CR20] Cardeña Etzel, Lynn Steven, Krippner Stanley (2014). Varieties of Anomalous Experience: Examining the Scientific Evidence.

[CR21] Cardeña Etzel, Van Duijl Marjolein, Weiner Lupita, Terhune Devin, Dell Paul, O’Neil John (2009). Possession/Trance Phenomena. Dissociation and the Dissociative Disorders: DSM-V and Beyond.

[CR22] CIA 2014 The World Factbook. Retrieved May 13, 2014, from https://www.cia.gov/library/publications/the-world-factbook/geos/dr.html.

[CR23] CLADEM 2008 Monitoreo sobre feminicidio/femicidio en República Dominicana. Retrieved July 24, 2014, from http://www.cladem.org/images/archivos/investigaciones/nacionales/rep-dominicana/Feminicidio-Rep-dom-2008.pdf.

[CR24] Crapanzano Vincent, Crapanzano Vincent, Garrison Vivian (1977). Introduction. Case Studies in Spirit Possession.

[CR25] Dalenberg Constance, Brand Bethany, Gleaves David, Dorahy Martin, Loewenstein Richard, Cardeña Etzel, Carlson Eve, Frewen Paul, Spiegel David (2012). Evaluation of the Evidence for the Trauma and Fantasy Models of Dissociation. Psychological Bulletin.

[CR26] Davis Martha Ellen (1987). La otra ciencia: El Vodú Dominicano como religion y medicina popular.

[CR27] Deive, Carlos 1996 [1975] Vodú y magia en Santo Domingo (Museo del Hombre Dominicano). Santo Domingo: Taller.

[CR28] Desmangles Leslie, Cardeña Etzel, van Quekelberghe Renault, Eigner Dagmar, Andritzky Walter (1994). Trance Possession and Vodou Ritual in Haiti. Yearbook of Cross-Cultural Medicine and Psychotherapy. Trance, Possession, Healing Rituals and Psychotherapy.

[CR29] Espirito Santo Diana, Hunter Jack, Luke David (2014). Developing the Dead in Cuba: An Ethnographic Account of the Emergence of Spirits and Selves in Havana. Talking with the Spirits. Ethnographies from Between the Worlds.

[CR30] Ferrándiz Francisco (2009). Open Veins. Spirits of Violence and Grief in Venezuela. Ethnography.

[CR31] Frigerio Alejandro (1989). Levels of Possession Awareness in Afro-Brazilian Religions. AASC Quarterly.

[CR32] Gaw Albert, Ding Qin-Zhang, Levine Ruth, Gaw Hsiao-Feng (1998). The Clinical Characteristics of Possession Disorder Among 20 Chinese Patients in the Hebei Province of China. Psychiatric Services.

[CR33] Glaser Barney, Strauss Anselm (2005). The Discovery of Grounded Theory: Strategies for Qualitative Research.

[CR34] Goff Donald, Brotman Andrew, Kindlon Daniel, Waites Meredith, Amico Edward (1991). The Delusion of Possession in Chronically Psychotic Patients. Journal of Nervous and Mental Disease.

[CR35] Hilgard Josephine (1970). Personality and Hypnosis. A Study of Imaginative Involvement.

[CR36] Hinton Devon, Lewis-Fernández Roberto (2010). Idioms of Distress among Trauma Survivors: Subtypes and Clinical Utility. Culture, Medicine and Psychiatry.

[CR37] Igreja Victor, Dias-Lambranca Beatrice, Hershey Douglas, Racin Limore, Richters Annemiek, Reis Ria (2010). The Epidemiology of Spirit Possession in the Aftermath of Mass Political Violence in Mozambique. Social Science & Medicine.

[CR38] Janet, Pierre 1976 [1919] Psychological Healing, vol. 1. New York: Arno Press.

[CR39] Lambek Michael, Ward Colleen (1989). From Disease to Discourse: Remarks on the Conceptualization of Trance and Spirit Possession. Altered States of Consciousness and Mental Health: A Cross-Cultural Perspective.

[CR40] Laria, Amaro 1998 Dissociative Experiences among Cuban Mental Health Patients and Spiritist Mediums. Dissertation. University of Massachusetts, Boston: Harvard Medical School.

[CR41] Lescot, Anne, and Laurence Magloire 2002 Des hommes et des deux. Documentary, Haiti/France, 52 min. (http://www.imdb.com/title/tt0391059/).

[CR42] Lewis, Ioan 1989 [1971] Ecstatic Religion. A Study of Shamanism and Spirit Possession. (2nd ed.) London: Routledge.

[CR43] Lewis-Fernández Roberto, Martínez-Taboas Alfonso, Sar Vedat, Patel Sapana, Boatin Adeline, Wilson John, Tang So-Kum (2007). Cross-Cultural Assessment of Psychological Trauma and PTSD. Cross-Cultural Assessment of Psychological Trauma and PTSD.

[CR44] López Irene, Ramirez Rafael, Guarnaccia Peter, Canino Gloriosa, Bird Hector (2011). Ataque de Nervios and Somatic Complaints among Island and Mainland Puerto Rican Children. CNS Neuroscience & Therapeutics.

[CR45] Maraldi Everton de Oliveira (2014). Medium or Author? A Preliminary Model Relating Dissociation, Paranormal Belief Systems and Self-Esteem. Journal of the Society for Psychical Research.

[CR47] Métraux Alfred (1955). Dramatic Elements in Ritual Possession. Diogenes.

[CR48] Moreira-Almeida Alejandro, Lotufo Neto Francisco, Greyson Bruce (2007). Dissociative and Psychotic Experiences in Brazilian Spiritist Mediums. Psychotherapy and Psychosomatics.

[CR49] Moreira-Almeida Alejandro, Lotufo Neto Francisco, Cardeña Etzel (2008). Comparison between Brazilian Spiritist Mediumship and Dissociative Identity Disorder. Journal of Nervous and Mental Disease.

[CR50] Negro Paulo, Palladino-Negro Paula, Louzã Mario (2002). Do Religious Mediumship Dissociative Experiences Conform to the Sociocognitive Theory of Dissociation?. Journal of Trauma & Dissociation.

[CR51] Neuner Frank, Pfeiffer Anett, Schauer-Kaiser Elisabeth, Odenwald Michael, Elbert Thomas, Ertl Verena (2012). Haunted by Ghosts: Prevalence, Predictors and Outcomes of Spirit Possession Experiences Among Former Child Soldiers and War-Affected Civilians in Northern Uganda. Social Science & Medicine.

[CR52] Ng Beng-Yeong (2000). Phenomenology of Trance States Seen at a Psychiatric Hospital in Singapore: A Cross-Cultural Perspective. Transcultural Psychiatry.

[CR53] Nijenhuis Ellert (2004). Somatoform Dissociation: Phenomena, Measurement, and Theoretical Issues.

[CR54] Nijenhuis Ellert (2010). The Scoring and Interpretation of the SDQ-20 and SDQ-5. Activitas Nervosa Superior.

[CR55] Nijenhuis Ellert, Spinhoven Philip, Van Dyck Richard, van der Hart Onno, Vanderlinden Johan (1996). The Development and Psychometric Characteristics of the Somatoform Dissociation Questionnaire (SDQ-20). The Journal of Nervous and Mental Disease.

[CR56] Nijenhuis Ellert, Spinhoven Philip, Van Dyck Richard, van der Hart Onno, Vanderlinden Johan (1997). The Development of the Somatoform Dissociation Questionnaire (SDQ-5) as a Screening Instrument for Dissociative Disorders. Acta Psychiatrica Scandinavica.

[CR57] Nijenhuis, Ellert, Onno van der Hart, and Johan Vanderlinden 2002 Cuestionario Sobre Disociaciones Somatoformes SDQ 20. Retrieved June 05, 2014, from http://www.enijenhuis.nl/sdq2.html.

[CR58] Nijenhuis Ellert, van der Hart Onno, Kruger Karlien (2002). The Psychometric Characteristics of the Traumatic Experiences Questionnaire (TEC): First Findings among Psychiatric Outpatients. Clinical Psychology & Psychotherapy.

[CR59] Oesterreich, Traugott 1974 [1921] Possession and Exorcism among Primitive Races, in Antiquity, the Middle Ages, and Modern Times. New York: Causeway. 10.1080/13648470.2016.1192874 27362545

[CR89] Ommeren Van, Mark B. Sharma, Ivan Komproe BN, Poudyal GK, Sharma Etzel Cardeña, de Jong JTVM (2001). Trauma and Loss as Determinants of Medically Unexplained Epidemic Illness in a Bhutanese Refugee Camp. Psychological Medicine.

[CR60] ONE 2009 Violencia conyugal en República Dominicana. Panorama Estadístico. (Boletín Mensual de la Oficina Nacional de Estadística, vol. 2). Santo Domingo.

[CR61] ONE, UNICEF, and OIT 2011 Dinámica del trabajo infantil en la República Dominicana. Encuesta Nacional de Hogares de Propósitos Múltiples ENHOGAR 2009–2010. Santo Domingo.

[CR62] OSAC 2013 Dominican Republic 2013. Crime and Safety Report. Retrieved July 11, 2013, from https://www.osac.gov/pages/ContentReportDetails.aspx?cid=14200.

[CR63] Owen Aex (1990). The Darkened Room: Women, Power, and Spiritualism in Late Victorian England.

[CR64] Pichler, Adelheid 2010 Havanna -Texturen und Bilder. Soziokulturelle Eigenlogiken der kubanischen Hauptstadt. Dissertation, Vienna: Universität Wien.

[CR65] Piper, Daniel Clifford 2012 Urbanization, Gender, and Cultural Emergence in the Music of Dominican Popular Religion: Palos and Salves in San Cristóbal, Dissertation, Providence: Brown University.

[CR100] Rouget, Gilbert 1985 Music and Trance. A Theory of the Relations between Music and Possession, Chicago: Chicago University Press

[CR66] Sapkota Ram P, Gurung Dristy, Neupane Deepa, Shah Santosh K, Kienzler Hanna, Kirmayer Laurence J (2014). A Village Possessed by “Witches”: A Mixed-Methods Case-Control Study of Possession and Common Mental Disorders in Rural Nepal. Culture, Medicine, and Psychiatry.

[CR67] Sar Vedat, Kundakci Turgut, Kiziltan Emre, Bakim Bahadir, Bozkurt Oya (2001). Differentiating Dissociative Disorders from other Diagnostic Groups through Somatoform Dissociation in Turkey. Journal of Trauma & Dissociation.

[CR68] Sar, Vedat, Firdevs Alioğlu, Gamze Akyüz 2014 Experiences of Possession and Paranormal Phenomena among Women in the General Population: Are they Related to Traumatic Stress and Dissociation? Journal of Trauma & Dissociation, 15(3), 303–318. 10.1080/15299732.2013.849321 24228817

[CR69] Schaffler, Yvonne 2009a Diagnose “Wolfspferd”. Spontanbesessenheiten in der Dominikanischen Republik als Anstoß für den Werdegang zum Heiler/zur Heilerin. Anthropos 104(2): 445–456.

[CR70] Schaffler, Yvonne 2009b Vodú? Das ist Sache der anderen! Kreolische Medizin, Spiritualität und Identität im Südwesten der Dominikanischen Republik (Wiener Ethnomedizinische Reihe). Wien - Zürich: LIT-Verlag.

[CR71] Schaffler Yvonne (2012). Besessenheit in der Dominikanischen Republik im Frühstadium: “Wilde” Besessenheit (caballo lobo) aus psychodynamischer und praxistheoretischer Perspektive. Curare.

[CR72] Schaffler Yvonne, Sigl Eveline, Schaffler Yvonne, Ávila Ricardo (2013). El caballo que se volvió lobo. Análisis del fenómeno de “posesión espontánea”. Etnografías de América Latina. Ocho Ensayos.

[CR73] Schaffler Yvonne, Felbeck Christine, Andre Klump (2015). “Wild” Spirit Possession in the Dominican Republic: From Expression of Distress to Cultural Expertise. “Dominicanidad” Schriftenreihe America Romana.

[CR74] Schmidt Bettina, Schmidt Bettina, Huskinson Lucy (2010). Possessed Women in the African Diaspora: Gender Difference in Spirit Possession Rituals. Spirit Possession and Trance: New Interdisciplinary Perspectives.

[CR75] Shweder Richard, Lessa William A, Vogt Evan Z (1972). Aspects of Cognition in Zinacanteco Shamans: Experimental Results. Reader in Comparative Religion: An Anthropological Approach.

[CR76] Seligman Rebecca, Kirmayer Laurence (2005). Distress, Dissociation, and Embodied Experience: Reconsidering the Pathways to Mediumship and Mental Health. Ethos.

[CR77] Seligman Rebecca, Kirmayer Laurence (2005). From Affliction to Affirmation: Narrative Transformation and the Therapeutics of Candomble Mediumship. Transcultural Psychiatry.

[CR80] Seligman Rebecca, Kirmayer Laurence (2008). Dissociative Experience and Cultural Neuroscience: Narrative, Metaphor and Mechanism. Culture, Medicine and Psychiatry.

[CR81] Somasundaram Daya, Thivakaran T, Bugra Dinesh (2008). Possession States in Northern Sri Lanka. Psychopathology.

[CR82] Spiegel David, Cardeña Etzel (1991). Disintegrated Experience: The Dissociative Disorders Revisited. Journal of Abnormal Psychology.

[CR83] Spiegel David, Loewenstein Richard, Lewis-Fernández Roberto, Sar Vedat, Simeon Daphne, Vermetten Eric, Cardeña Etzel, Dell Paul (2011). Dissociative Disorders in DSM-5. Depression and Anxiety.

[CR84] U.S. Department of State 2005 International Religious Freedom Report. Retrieved May 13, 2014, from http://www.state.gov/j/drl/rls/irf/2005/51636.htm.

[CR85] Van der Port Mattijs (2005). Candomblé in Pink, Green and Black. Re-scripting the Afro-Brazilian Religious Heritage in the Public Sphere of Salvador, Bahia. Social Anthropology.

[CR87] Van Duijl Marjolein, Nijenhuis Ellert, Komproe Ivan, Gernaat Hajo, de Jong Joop (2010). Dissociative Symptoms and Reported Trauma among Patients with Spirit Possession and Matched Healthy Controls in Uganda. Culture, Medicine and Psychiatry.

[CR86] Van Duijl Marjolein, Cardeña Ezel, de Jong Joop (2005). The Validity of DSM-IV Dissociative Disorders Categories in South-West Uganda. Transcultural Psychiatry.

[CR88] Van Duijl Marjolein, Kleijn Wim, de Jong Joop (2013). Are Symptoms of Spirit Possessed Patients Covered by the DSM-IV or DSM-5 Criteria for Possession Trance Disorder? A Mixed-Method Explorative Study in Uganda. Social Psychiatry and Psychiatric Epidemiology.

[CR90] Ward Colleen, Beaubrun Michael (1981). Spirit Possession and Neuroticism in a West Indian Pentecostal Community. British Journal of Clinical Psychology.

[CR91] Wimmer, August 1993 [1924] Om Besaettelser (On Possession States). History of Psychiatry 4(15): 420–440.

